# Development of aminoglycoside and β-lactamase resistance among intestinal microbiota of swine treated with lincomycin, chlortetracycline, and amoxicillin

**DOI:** 10.3389/fmicb.2014.00580

**Published:** 2014-11-04

**Authors:** Jian Sun, Liang Li, Baotao Liu, Jing Xia, Xiaoping Liao, Yahong Liu

**Affiliations:** Laboratory of Veterinary Pharmacology, College of Veterinary Medicine, South China Agricultural UniversityGuangzhou, China

**Keywords:** culture-independent method, qPCR, antimicrobial-resistant genes, 16S *rRNA*, Bacterial Enumeration

## Abstract

Lincomycin, chlortetracycline, and amoxicillin are commonly used antimicrobials for growth promotion and infectious disease prophylaxis in swine production. In this study, we investigated the shifts and resistance development among intestinal microbiota in pregnant sows before and after lincomycin, chlortetracycline, and amoxicillin treatment by using phylogenetic analysis, bacterial enumeration, and PCR. After the antimicrobial treatment, shifts in microbial community, an increased proportion of resistant bacteria, and genes related to antimicrobial resistance as compared to the day before antimicrobial administration (day 0) were observed. Importantly, a positive correlation between antimicrobial resistance gene expression in different categories, especially those encoding aminoglycoside and β-lactamase and antimicrobial resistance, was observed. These findings demonstrate an important role of antimicrobial usage in animals in the development of antimicrobial resistance, and support the notion that prudent use of antimicrobials in swine is needed to reduce the risk of the emergence of multi-drug resistant zoonotic pathogens.

## INTRODUCTION

During animal production, antimicrobials are routinely used at subtherapeutic levels for growth promotion and infectious disease prevention, as well as at therapeutic levels for treatment of infections (Jindal et al., 2006). Various antimicrobials have made significant contributions to lowered incidence of diseases, reducing morbidity and mortality, and for production of abundant quantities of nutritious, high-quality, low-cost food for human consumption (Oliver et al., 2011). For the year ending December 2007, global sales of animal health products (including antimicrobials) totaled $17.9 billion, which increased tremendously from $8.65 billion in 1992 (Page and Gautier, 2012).

Although there are significant benefits of antimicrobials in agriculture, liberal antimicrobial use has raised lasting concerns about antimicrobial residues in animal food products, and a rapid and widespread emergence of resistance to multiple antimicrobials among both animal and human pathogens (Aarestrup and Wegener, 1999). Furthermore, the antimicrobial residues and antimicrobial-resistant bacteria animal feces are potential “time bombs,” because they could be excreted into the environment and threaten public health for a long time (Mathew et al., 1998). To make things worse, antimicrobial combinations are used most frequently to provide a broad-spectrum of antimicrobial activity, or in an attempt to promote better growth performance in food-producing animals. Although negative effects – such as development of changes in commensal microorganisms, as well as the emergence of an enormous diversity of antimicrobial resistance genes and multidrug-resistant strains in animals – have been ignored for a long time, they have attracted more and greater attention in recent years (Hao et al., 2014).

Using high-throughput metagenomics, Looft et al. (2012) revealed that the bacterial phylotypes shifted after antimicrobial treatment, and the microbial functional genes relating to energy production and conversion were increased in antimicrobial-fed pigs. The findings shed light on the fact that the addition of antimicrobials to animal feed introduces a selective pressure that may lead to lasting changes among commensal livestock microorganisms. It has also been demonstrated that the use of antimicrobials may promote the development of antimicrobial resistance among bacteria in animals (Singer et al., 2003). Usually, there is a positive correlation between administration of antimicrobials and fecal counts of corresponding resistant bacteria. For instance, administration of fluoroquinolone could promote the emergence of quinolone-resistant *Enterobacteria* in the fecal flora of pigs (Nguyen et al., 2012). In addition, use of a single antimicrobial may induce cross-resistance to other antimicrobials that were not administrated. Receiving a diet containing chlortetracycline, sulfamethazine, and penicillin was associated with a rise of aminoglycoside resistance in pigs (Looft et al., 2012). In fact, horizontal gene transfer is largely the cause of the multi-drug resistance phenotype in intestinal bacteria. The mobile genetic element harboring antimicrobial-resistance genes (ARGs) can transfer among different gastrointestinal bacteria, which could be accelerated with the selective pressure of antimicrobials *in vivo* (van Essen-Zandbergen et al., 2009).

There have been various strategies for investigating livestock microbiota and fecal ARGs. Valuable information has been obtained by culture-based approaches to investigate narrow groups of bacteria or genes isolated from animal feces, such as cephalosporin resistance in cattle isolates (Madec et al., 2008). Recently, a culture-independent method that utilizes extracted DNA from all microorganisms present in fecal samples has been widely used. This technique provides a global view of the bacterial community, and assists in predicting ecosystem functioning and further understanding of bacterial evolution (Roesch et al., 2009; Looft et al., 2012). There is a paucity of data with regard to the effects of subtherapeutic doses of antimicrobials on shifts and antimicrobial resistance in intestinal microbiota, as well as levels of various ARGs.

In the present study, we investigated phylogenetic clustering of the intestinal microbiota, bacterial communities resistant to antimicrobials, and levels of ARG expression before and after a single administration of subtherapeutic doses of antimicrobials to swine.

## MATERIALS AND METHODS

### SWINE, TREATMENT, AND SAMPLING

A pen (24 pregnant sows) from a typical intensive swine production farm (2400 sows) in Guangdong Province, China, was selected for the study. After artificial insemination, 24 sows were fed the same diet without antimicrobials for at least 90 days. This withdrawal period minimized the effects of drug residues on their gastrointestinal microbiota (Arikan et al., 2006). In order to prevent postpartum diseases, farrowing sows were given the above diet containing lincomycin hydrochloride (500g/ton), chlortetracycline (100g/ton), and amoxicillin (500g/ton) before delivery. Fresh fecal samples were collected from sows at 0 days before treatment (defined as A) as the baseline (single administration of subtherapeutic doses of the antimicrobials) and 3, 6, and 12 days after treatment (B, C, and D). At each point, nine fecal samples were randomly collected from 24 sows, and were then pooled to form a single composite sample. The study protocol was reviewed and approved by the South China Agriculture University animal ethics committee. The owners of the sows from which fecal samples were taken gave permission for their animals to be used in this study.

### DNA EXTRACTIONS

Total DNA from fecal samples was extracted as previously described Looft et al. (2012). DNA samples were quantified *via* BioPhotometer plus (Eppendorf, Shanghai, China) and gel electrophoresis. Extracted DNA samples had an A260/280 ratio of ≥1.6, and an A260/230 ratio of ≥2.0.

### AMPLIFICATION AND 454 SEQUENCING OF THE 16S *rRNA* GENE

The V3-V5 regions of the bacterial *16S rRNA* gene were amplified with conserved primers: 357F (V3 primer) 5′-CCTACGGGAGGCAGCAG-3′ and 926R (V5 primer) 5′-CCG TCAATTCMTTTRAGT-3′ (Methe et al., 2012). Amplification primers were designed with FLX Titanium adapters (A adapter sequence: 5′-CCATCTCATCCCTGCGTGTCTCCGACTCAG-3′; B adapter sequence: 5′-CCTATCCCCTGTGTGCCTTGGCAGTC TCAG-3′), and a sample barcode sequence where applicable. Forward primers contained the B adapter, and the reverse primers contained the A adapter. PCR products were then sequenced using the Roche-454 FLX Titanium platform according to the “HMP 16S Protocol” (http://www.hmpdacc.org/doc/HMP_MDG_454_16S_Protocol.pdf), and Ward et al. (2012). After adoption of the *16S rRNA* protocol, including removal of multiple sources of potential artifacts or bias generated by *16S rRNA* sequencing using pyrosequencing (Huse et al., 2007), phylogenetic analysis and taxonomic assignments of the V3-V5 regions within the *16S rRNA* gene were performed using the mothur and QIIME (Schloss et al., 2009; Caporaso et al., 2010).

### BACTERIAL ENUMERATION

Six commonly used veterinary antimicrobials (ciprofloxacin, tetracycline, erythromycin, clindamycin, gentamycin, and ampicillin) were selected to estimate the relative abundance of antimicrobial-resistant bacteria in fecal samples. Bacterial were enumerated using a method described by (Yashiro and McManus, 2012); 3 g of the composite samples were blended in 50 ml sterile PBS. After suspension, the sample was serially diluted and then plated onto tryptic soy agar (TSA), as well as TSA amended with ciprofloxacin (4 μg/ml), tetracycline (16 μg/ml), erythromycin (8 μg/ml), clindamycin (4 μg/ml), gentamicin (16 μg/ml), and ampicillin (32 μg/ml) to select for each antimicrobial-resistant bacteria. After incubation for 7 days at room temperature (Yashiro and McManus, 2012), colony-forming units (cfu) were counted, and the proportion resistant to antimicrobials was calculated. The proportion of bacterial communities resistant to each antimicrobial of sample A was treated as the control. Bacterial enumeration assays were performed on three separate experiments, with three replications *per* assay used for each experiment.

### DETECTION AND RELATIVE QUANTIFICATION OF ARGs

Polymerase chain reaction (PCR) detection assays were used to screen six ARG types: plasmid-mediated quinolone resistance (PMQR), tetracycline, macrolide, lincomycin, aminoglycoside inactivating enzymes, and β-lactamase in the fecal samples. All primers used in this study are shown in Table S1. To ensure reproducibility, three replicate assays for each sample were performed in parallel with positive and negative controls in each run. ARGs detected by PCR and *16S rRNA* genes were further quantified by qPCR using the SybrGreen approach. The qPCR was performed with SYBR® Premix Ex Taq^TM^ (TaKaRa Bio) in a thermal cycler (iQ5; Bio-Rad, Hercules, CA, USA) according to the manufacturer’s instructions. The conditions were as follows: denaturing at 94°C for 5 min, followed by 35 cycles at 94°C for 1 min, at 60°C for 1 min, and at 72°C for 1 min, with a final extension at 72°C for 5 min; the melt curve was read from 60 to 95°C every 1°C at the end of the assay. A Eubacterial 16S *rRNA* gene was also quantified according to the SybrGreen approach (Bach et al., 2002), so that ARGs could be normalized to the total bacterial community. This step provided a means to correct for potential variations in extraction efficiencies and compare ARGs proportionally between samples of different overall population sizes. All DNA samples were diluted to 2 ng/μL in TE buffer prior to qPCR amplification. Matrix effects associated with extraction of DNA from fecal samples were corrected as previously described (Pei et al., 2006). The ARG copy numbers normalized to ambient *16S rRNA* gene copies of sample A were treated as the control. All experiments were performed in triplicate, and standard error of the measurements was determined from these parallel data.

## STATISTICAL ANALYSIS

Data analysis was conducted using SPSS version 16.0. A two-tailed Student’s *t*-test was calculated on the mean percentage of antimicrobial resistance between the samples to determine the impact of single administration of subtherapeutic doses of antimicrobials on bacterial populations. A p-value of <0.05 was considered statistically significant. Changes in ARG copy numbers were considered to be significant when the corresponding ratios were ≥2.5 or ≤0.4. A two-tailed Pearson’s bivariate correlation analysis was used to compare relative abundances of ARGs.

## RESULTS

### SHIFTS IN MICROBIAL COMMUNITY WITH ANTIMICROBIAL TREATMENT

**FIGURE 1:**
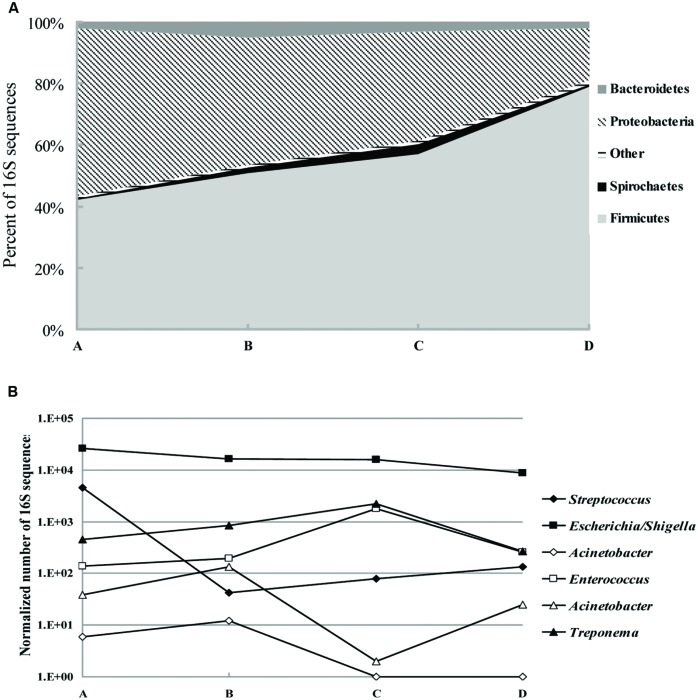
**Shifts in fecal bacterial community memberships with antibiotic treatment. (A)** Phylum-level composition of fecal microbial communities. Data were pooled for fecal samples isolated from the day 0 before treatment (A) and the day 3, 6, 12 after treatment (B–D) and are shown as percentage of abundance. **(B)** Genus-level composition of common pathogens and opportunistic pathogens, shown as the total number of sequences (normalized to 50000 total reads).

We collected 79194 sequences of the V3–V5 regions of bacterial 16S *rRNA* genes from a total of five fecal samples. The majority of classifiable sequences (95.6 to 98.6%) belonged to the *Firmicutes*, Proteobacteria, and *Bacteroidetes* phyla. Specific changes in the microbial community associated with antimicrobial treatment included a decrease from 54.6 to 16.8% (*p* < 0.005) in the abundance of *Proteobacteria* phyla. In addition, the increase in *Firmicutes* abundance with antimicrobial treatment was particularly striking, with 42.1% of the population in sample A to 79% of the population in sample D (*p* < 0.05; **Figure 1A**). Members of the *Bacteroidetes*, *Spirochaetes*, *Euryarchaeota*, and *Actinobacteria* phyla increased by 2.5-, 5-, 1.1-, and 2-fold, respectively, (*p* < 0.05) at the third or sixth day after antimicrobial treatment (Sample B or C), and then returned to normal levels 12 days after treatment. Genus-level composition of common pathogens and opportunistic pathogens indicated that after antimicrobial treatment *Escherichia*/*Shigella* decreased by 40% (*p* < 0.05). Interestingly, *Streptococcus* populations were the major difference before and after treatment: 11–110-fold less abundance in samples B, C, and D than sample A (*p* < 0.05; **Figure 1B**). *Treponema*, *Enterococcus*, and *Staphylococcus* populations were increased by 6-, 14-, 2-fold, respectively, (*p* < 0.05) within a week but returned to baseline 6 days later.

### SHIFTS IN THE NUMBER OF RESISTANT BACTERIA WITH ANTIMICROBIAL TREATMENT

**FIGURE 2:**
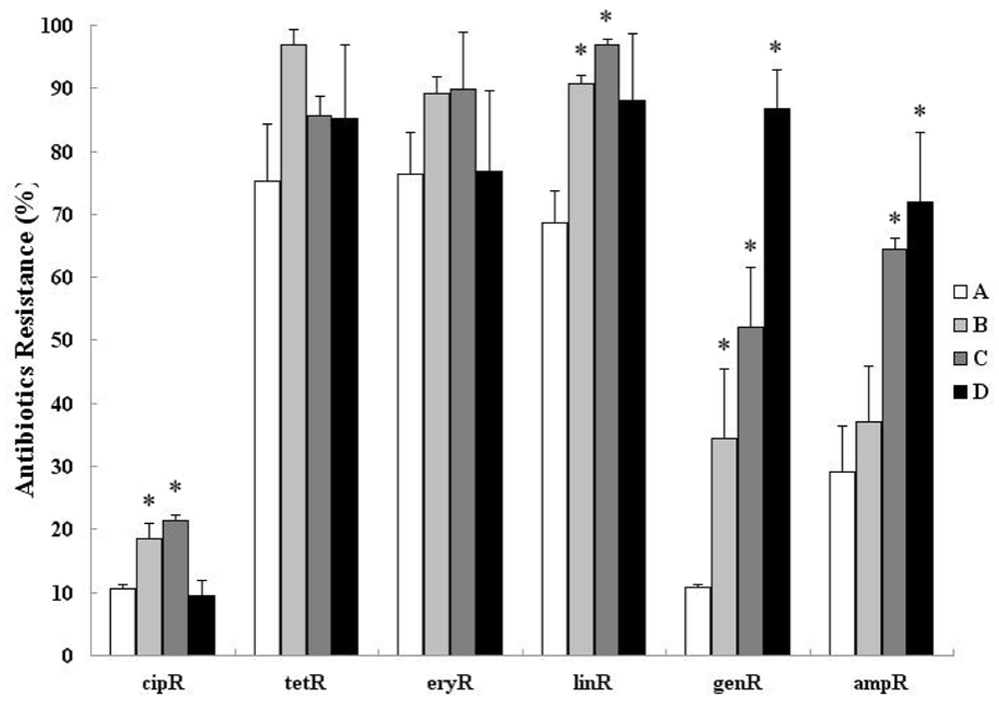
**Cultivation based estimation of relative abundance of ciprofloxacin-resistant (cipR), tetracycline-resistant (tetR), erythromycin-resistant (eryR), clindamycin-resistant (linR), gentamycin-resistant (genR), and ampicillin-resistant (ampR) bacteria in fecal samples isolated from day 0 before treatment (A) and day 3, 6, 12 after treatment (B–D).** Asterisks indicate statistically significant (*p* < 0.05) differences between day 0 before treatment and day 3, 6, 12 after treatment.

The proportion of bacteria communities resistant to tetracycline, erythromycin, and clindamycin were increased after treatment. Only clindamycin-resistant bacterial communities were increased significantly (*p* < 0.01; **Figure 2**). Moreover, the proportion of bacterial communities resistant to gentamycin and ampicillin still shows an upward trend (from 20.4 and 29.6 to 87.1% and 73.8%, respectively, – *p* < 0.001) 12 days after treatment (**Figure 2**). An increase in the proportion of bacterial communities that was resistant to ciprofloxacin was observed after treatment (from 10.4 to 18.8 to 21.3% – *p* < 0.05), and it was then recovered in sample D collected on day 12 (9.8%).

### SHIFTS IN ARGs ABUNDANCE WITH ANTIMICROBIAL TREATMENT

**FIGURE 3:**
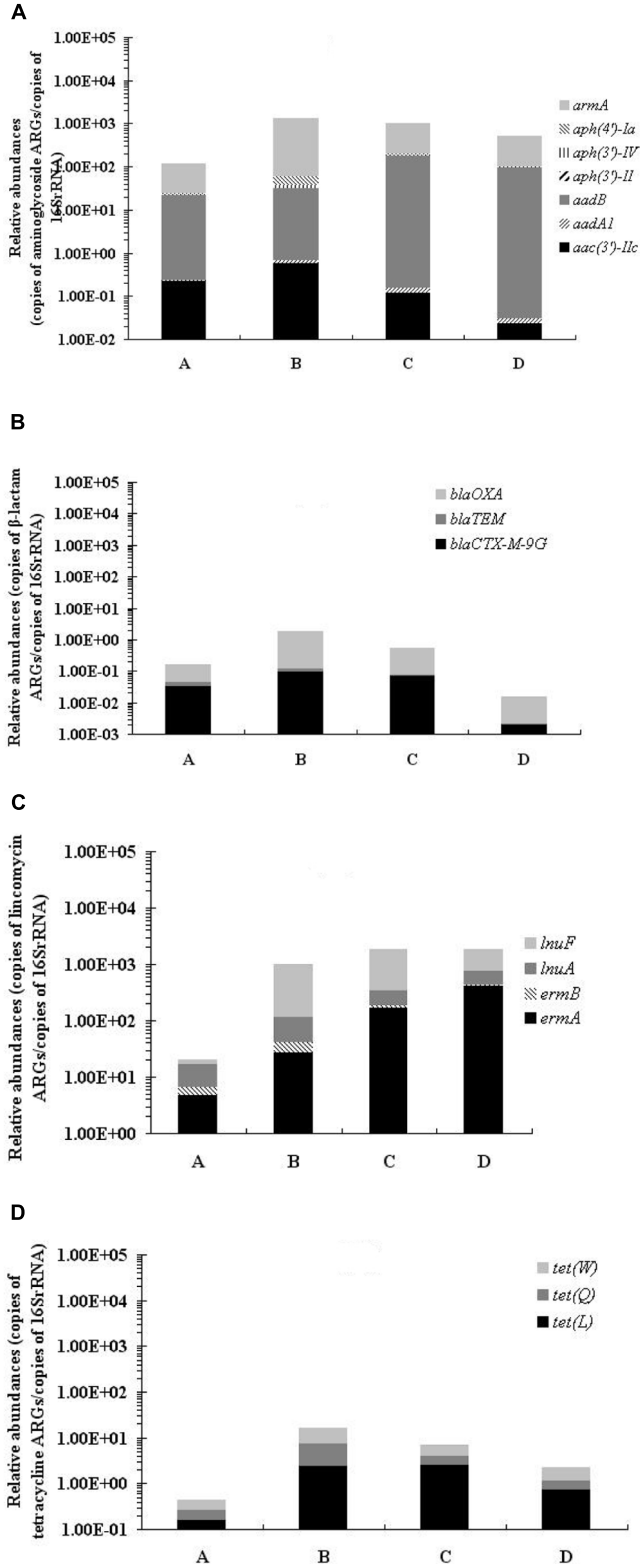
**Relative abundances of aminoglycoside ARGs (A), β-lactam ARGs (B), lincomycin ARGs, (C) and tetracycline ARGs (D) in fecal samples isolated before and after treatment, normalized to ambient 16S *rRNA* gene copies**.

Among the 53 ARGs investigated, PMQR ARGs: *aac(6′)-Ib-cr* and *qepA*, tetracycline ARGs: *tet*(L), *tet*(Q) ,and *tet*(W), macrolide ARGs: *ermA*, *ermB*, *mefA,* and *ereA*, lincomycin ARGs: *ermA*, *ermB*, *lnuA,* and *lnuF*, aminoglycoside inactivating enzyme encoding ARGs: *aac(3′)-IIc*, *aadA1*, *aadB*, *aph(3′)-II*, *aph(3′)-IV*, *aph(4′)-Ia,* and *armA*, and β-lactamase-encoding ARGs: *bla*_TEM_, *bla*_CTX-M-9G_ and *bla*_OXA_ were detected in all samples before and after treatment. In general, the qPCR results revealed 4 ARG types with significantly greater abundance (by 10-, 50-, 1000-, and 40-fold, respectively, *p* < 0.01) in samples collected after treatment than before treatment: aminoglycoside ARGs, lincomycin ARGs, macrolide ARGs, and tetracycline ARGs (**Figure 3**; Table S2). The relative abundance of β-lactam ARGs increased at first, but then decreased after treatment.

### CORRELATION ANALYSIS

Significant, positive correlations among different resistance genes in the same category of ARGs were observed in the relative abundances of 6 ARG types (Table S3). Furthermore, there was a significant correlation between various types of ARGs, such as the sum of macrolide ARGs, sum of aminoglycoside ARGs (*r* = 0.945, *p* = 0.015), and sum of β-lactam ARGs (*r* = 0.961, *p* = 0.009).

## DISCUSSION

We assessed the shifts in fecal microbiota in sows that received antimicrobial treatment. The ratio of *Bacteroidetes* to *Firmicutes* decreased significantly 12 days after antimicrobial treatment. Previous studies reported that obese mice had a lower ratio of *Bacteroidetes* to *Firmicutes* in their feces compared with lean mice. Presumably because of this shift, obese mice have an improved energy-harvesting capacity (Turnbaugh et al., 2006). Perhaps growth-promoting benefits obtained from feeding swine antimicrobials, especially chlortetracycline, as part of their diets are due to this shift. Additionally, *Escherichia coli* is a common porcine enteric pathogen, causing diarrhea in new-born and weaned pigs, and edema in pigs after weaning (Frydendahl et al., 2003). Antimicrobials could decrease the duration of fecal shedding of *E. coli* by swine (Cornick, 2010), and therefore the decrease in *E. coli* following antimicrobial treatment could help reduce the risk of transmission from sows to piglets, thus reducing the morbidity and mortality of piglets. In this study, a decrease (40%, *p* < 0.05) in *Escherichia/Shigella* was found after treatment with antimicrobials. The same beneficial decline was also found in *Streptococcus* in our study, among which *Streptococcus suis* is mainly associated with bronchopneumonia, meningitis, and septicemia (Higgins et al., 1990). However, other pathogens or opportunistic pathogens such as *Staphylococcus aureus*, *Treponema hyodysenteriae*, and *Enterococcus faecalis* increased several days after antimicrobial treatment. These increases would be an undesirable collateral effect of in-feed antimicrobials, and are a threat to the health of sows and piglets (Larson et al., 1985). Moreover, these potential pathogens may eventually threaten human health through the food chain.

In this study, antimicrobial treatment caused a detectable increase in the proportion of antimicrobial-resistant bacteria in the community and the abundance of resistance genes. Many of these resistance genes were likely enriched because of direct interaction with the antimicrobials such as tetracycline, clindamycin, and ampicillin. Tetracycline is used frequently for the treatment and/or prevention of bacterial diseases and for growth promotion within the swine industry (Agriculture, 1996, 2000). Furthermore, a low, short-term dose of in-feed tetracycline could promote an increase in tetracycline efflux pump expression (Looft et al., 2012). Therefore, the tetracycline resistance rate could remain at a high level before treatment and increase from 69.8 to 75.4 to 85.4% to 97.1% after treatment in this study. Viable but non-culturable (VBNC) bacteria were not considered in the bacterial enumeration assay under the culture condition described in the method, because they accounted for only a few of each zoonotic pathogen (Pinto et al., 2011; Trevors, 2011).

An increase in the abundance of the sum of tetracycline ARGs was detected after in-feed antimicrobial treatment. A similar situation also appears with clindamycin. In this study, a high level of *erm*(A) and *erm*(B) genes, which could confer resistance to both clindamycin and macrolide, was detected before and after treatment. Additionally, decreased cell membrane permeability and/or multi-drug efflux pumps could make macrolides ineffective against gram-negative bacteria. These two factors may have contributed to the high macrolide-resistance rates in this study (Levy, 2002; Rodrigues et al., 2009).

It is noteworthy that the aminoglycoside ARGs increased in abundance with treatment, although they do not confer resistance to the antimicrobials therein. Furthermore, significant correlations among different category ARGs, such as *bla*_OXA_ and *aadA1*, *bla*_OXA,_ and *aac(3′)-IIc* were observed. This might be explained by the previous findings that *bla*_OXA-21_ and *aadA1* could both be embedded within a class 1 integron in a gene cassette array, and *bla*_OXA_ and *aac(3′)-IIc* genes could be co-located on the same large conjugative IncFII(K) plasmids (Dolejska et al., 2012; Yamamoto et al., 2013). Additionally, *tet* and *aadA1*, which could co-locate in the same strains, also presented significant correlations (Radhouani et al., 2013). Overall, due to the indirect mechanism of selection mentioned above, the observation of an increased abundance in aminoglycoside ARGs after treatment in our study can be partially explained. We also observed the correlation between different category ARGs, such as *mefA* and *armA*, and *ereA* and *armA*. Although reports of these resistance genes located in the same bacteria or mobile elements are lacking, we remain concerned about these genes which could be indirectly induced by antimicrobials, and might cause the emergence of MDR bacteria. More so, the ARGs remain at a high level 12 days after treatment, and could be continually discharged into the environment, thus may potentially threaten public health.

In conclusion, these results indicate that even a low, short-term dose of antimicrobials can increase the proportion of resistant-bacterial communities, abundance of ARGs, including resistance to antimicrobials not administered, and the abundance of potential human pathogens, such as *S. aureus*. Additionally, the positive correlation between different category ARGs showed that the emergence of MDR bacteria might be due to the collateral effects of antimicrobial use. The data from this study further warn that the prudent use of antimicrobials in swine is necessary to reduce the risks of emergence of multi-drug resistance zoonotic pathogens.

## Conflict of Interest Statement

The authors declare that the research was conducted in the absence of any commercial or financial relationships that could be construed as a potential conflict of interest.
