# Supplementary material for: Development of aminoglycoside and β-lactamase resistance among intestinal microbiota of swine treated with lincomycin, chlortetracycline, and amoxicillin
Source: Front Microbiol. 2014 Nov 4;5:580. doi: 10.3389/fmicb.2014.00580 (PMC4219486; doi:10.3389/fmicb.2014.00580)
Supplement: Supplementary file 1 [file Table_1.DOC]

**Table S1.** PCR (QPCR) primers and conditions used in this study.

| *Gene* | *Oligonucleotide sequence (5’-3’)* | *Product size* | *Temperature* | *Reference* |
| --- | --- | --- | --- | --- |
| Quinolone ARGs |  |  |  |  |
| *qnrA* | AGAGGATTTCTCACGCCAGG | 580 bp | 54 °C |  |
|  | TGCCAGGCACAGATCTTGAC |  |  |
| *qnrB* | GGMATHGAAATTCGCCACTG | 264 bp | 54 °C |  |
|  | TTTGCYGYYCGCCAGTCGAA |  |  |
| *qnrC* | GGGTTGTACATTTATTGAATCG | 307 bp | 55 °C |  |
|  | CACCTACCCATTTATTTTCA |  |  |
| *qnrD* | CGAGATCAATTTACGGGGAATA | 465 bp | 50 °C |  |
|  | AACAAGCTGAAGCGCCTG |  |  |
| *qnrS* | GCAAGTTCATTGAACAGGGT | 428 bp | 54 °C |  |
|  | TCTAAACCGTCGAGTTCGGCG |  |  |
| *aac(6’)-Ib-cr* | TTGCGATGCTCTATGAGTGGCTA | 482 bp | 58 |  |
|  | CTCGAATGCCTGGCGTGTTT |  |  |  |
| *qepA* | CCAGCTCGGCAACTTGATAC | 570 bp | 60 °C |  |
|  | ATGCTCGCCTTCCAGAAAA |  |  |  |
| *oqxA* | CTCGGCGCGATGATGCT | 392 bp | 57 °C |  |
|  | CCACTCTTCACGGGAGACGA |  |  |
| Tetracycline ARGs |  |  |  |  |
| *tet*(A) | GCTACATCCTGCTTGCCTTC | 210 bp | 55 °C |  |
|  | CATAGATCGCCGTGAAGAGG |  |  |  |
| *tet*(C) | CTTGAGAGCCTTCAACCCAG | 418 bp | 55 °C |  |
|  | ATGGTCGTCATCTACCTGCC |  |  |  |
| *tet*(G) | GCTCGGTGGTATCTCTGCTC | 468 bp | 55 °C |  |
|  | AGCAACAGAATCGGGAACAC |  |  |  |
| *tet*(L) | TCGTTAGCGTGCTGTCATTC | 267 bp | 55 °C |  |
|  | GTATCCCACCAATGTAGCCG |  |  |  |
| *tet*(M) | ACAGAAAGCTTATTATATAAC | 171 bp | 45 °C |  |
|  | TGGCGTGTCTATGATGTTCAC |  |  |
| *tet*(O) | ACGGARAGTTTATTGTATACC | 171 bp | 45 °C |  |
|  | TGGCGTATCTATAATGTTGAC |  |  |
| *tet*(Q) | AGAATCTGCTGTTTGCCAGTG | 169 bp | 55 |  |
|  | CGGAGTGTCAATGATATTGCA |  |  |
| *tet*(W) | GAGAGCCTGCTATATGCCAGC | 168 | 60 |  |
|  | GGGCGTATCCACAATGTTAAC |  |  |
| *tet*(X) | CAATAATTGGTGGTGGACCC | 468 bp | 58 |  |
|  | TTCTTACCTTGGACATCCCG |  |  |  |
| Lincomycin ARGs |  |  |  |  |
| *lnuA* | GGTGGCTGGGGGGTAGATGTATTAACTGG | 323 | 57 |  |
|  | GCTTCTTTTGAAATACATGGTATTTTTCGA |  |  |  |
| *lnuB* | CCTACCTATTGTTTGTGGAA | 925 | 54 |  |
|  | ATAACGTTACTCTCCTATTTC |  |  |
| *lnuC* | AATTTGCAATAGATGCGGAGA | 1100 | 55 |  |
|  | TCATGTGCATTTTCATCA |  |  |
| *lnuD* | ACGGAGGGATCACATGGTAA | 475 | 56 |  |
|  | TCTCTCGCATAATAACCTTACGTC |  |  |
| *lnuF* | CACCATGCTTCAGCAGAAAATGATC | 1200 | 55 |  |
|  | TTACTTGTTGTGCGGCGTC |  |  |
| *lsaA* | CGCTCCAGCTGTATGAGAACTGC | 1200 | 55 |  |
|  | TCAAGCGATTGACTTCTTTTTTG |  |  |
| *lsaB* | TGATATTGTCTCTTGGAAGG | 1100 | 56 |  |
|  | AATGAACGCTTGCAGAAGGA |  |  |
| *lsaC* | GGCTATGTAAAACCTGTATTTG | 429 | 55 |  |
|  | ACTGACAATTTTTCTTCCGT |  |  |
| Macrolide ARGs |  |  |  |  |
| *mefA* | AGTATCATTAATCACTAGTGC | 345 | 56 | This study |
|  | TTCTTCTGGTACTAAAAGTGG |  |  |  |
| *msrA* | GCAAATGGTGTAGGTAAGACAACT | 399 | 55 |  |
|  | TAAAACAAATGTAGTGTACTA |  |  |
| *msrD* | CCTTATCGGCACAGGTTCAT | 500 | 55 |  |
|  | GCCTTCCGGAGCTCCTACTT |  |  |
| *ereA* | AACACCCTGAACCCAAGGGACG | 420 | 50 |  |
|  | CTTCACATCCGGATTCGCTCGA |  |  |
| *ereB* | AGAAATGGAGGTTCATACTTACCA | 546 | 50 |  |
|  | CATATAATCATCACCAATGGCA |  |  |
| *mphA* | AACTGTACGCACTTGC | 837 | 50 |  |
|  | GGTACTCTTCGTTACC |  |  |
| *mphC* | GGGAAATTGAACACAAACC | 500 | 54 |  |
|  | AATTCATCTGATACACCATAAG |  |  |
| MLSB ARGs |  |  |  |  |
| *ermA* | GTTCAAGAACAATCAATACAGAG | 421 |  | This study |
|  | GGATCAGGAAAAGGACATTTTAC |  |  |  |
| *ermB* | GAAAAGGTACTCAACCAAATA | 639 |  | This study |
|  | AGTAACGGTACTTAAATTGTTTAC |  |  |  |
| *ermC* | TCAAAACATAATATAGATAAA | 642 |  |  |
|  | TAACTGCTAAATTTGTTATAATCG |  |  |
| *ermTR* | ACAGAAAAACCCCGAAAAATACG | 679 |  |  |
|  | TTGGATAATTTATCAAGATCAG |  |  |
| Aminoglycoside ARGs |  |  |  |  |
| *aac(3’)-Ia* | TTACGCAGCAGCAACGATGT | 402 | 58.5 | This study |
|  | GTTGGCCTCATGCTTGAGGA |  |  |  |
| *aac(3’)-IIc* | AACCGGTGACCTATTGATGG | 774 | 58.5 | This study |
|  | TGTGCTGGCACGATCGGAGT |  |  |  |
| *aadA1* | AGGTAGTTGGCGTCATCGAG | 589 | 58.5 | This study |
|  | CAGTCGGCAGCGACATCCTT |  |  |  |
| *aadB* | GCGAAATCTGCCGCTCTG | 412 | 58 | This study |
|  | TGCGAGCCTGTAGGACTC |  |  |  |
| *aph(3’)-II* | TCTGAAACATGGCAAAGGTAG | 582 | 54 | This study |
|  | AGCCGTTTCTGTAATGAAGGA |  |  |  |
| *aph(3’)-IV* | AGAACGAGATGACGTTGGAG | 1037 | 56.5 | This study |
|  | AGTTGGTCAAGACCAATGCG |  |  |  |
| *aph(4’)-Ia* | TCCGGAAGTGCTTGACATTG | 540 | 58.5 | This study |
|  | GGATGCCTCCGCTCGAAGTA |  |  |  |
| *armA* | ATTCTGCCTATCCTAATTGG | 315 | 55 |  |
|  | ACCTATACTTTATCGTCGTC |  |  |
| *rmtA* | CTAGCGTCCATCCTTTCCTC | 635 | 55 |  |
|  | TTGCTTCCATGCCCTTGCC |  |  |
| *rmtB* | GCTTTCTGCGGGCGATGTAA | 173 | 55 |  |
|  | ATGCAATGCCGCGCTCGTAT |  |  |
| *rmtC* | CGAAGAAGTAACAGCCAAAG | 711 | 55 |  |
|  | ATCCCAACATCTCTCCCACT |  |  |
| *rmtD* | CGGCACGCGATTGGGAAGC | 401 | 55 |  |
|  | CGGAAACGATGCGACGAT |  |  |
| *aac(6’)-Ib* | TGCTGACGTACAGGAACAGT | 354 | 58 | This study |
|  | GTTACGGTACCTTGCCTCTC |  |  |  |
| β-lactam ARGs |  |  |  |  |
| *bla*_TEM_ | ATAAAATTCTTGAAGACGAAA | 1083 | 53 |  |
|  | GACAGTTACCAATGCTTAATC |  |  |
| *bla*_SHV_ | CACTCAAGGATGTATTGTG | 885 | 56 |  |
|  | TTAGCGTTGCCAGTGCTCG |  |  |
| *bla*_DHA_ | GTTACTCACACACGGAAGGT | 800 | 56 | This study |
|  | TTTTATAGTAGCGGGTCTGG |  |  |  |
| *bla*_CTX-M-1G_ | ATGGTTAAAAAATCACTGCGCC | 890 | 56 | This study |
|  | TCCCGACGGCTTTCCGCCTT |  |  |  |
| *bla*_CTX-M-2G_ | CTCAGAGCATTCGCCGCTCA | 843 | 56 | This study |
|  | CCGCCGCAGCCAGAATATCC |  |  |  |
| *bla*_CTX-M-9G_ | GCGCATGGTGACAAAGAGAGTGCAA | 876 | 56 | This study |
|  | GTTACAGCCCTTCGGCGATGATTC |  |  |  |
| *bla*_CTX-M-25G_ | GTAAGGGGGGGGATGTTAAT | 856 | 55 | This study |
|  | AACCGTCGGTGACAATTCTG |  |  |  |
| *bla*_CMY-2_ | ATGATGAAAAAATCGTTATGC | 1143 | 55 |  |
|  | TTGCAGCTTTTCAAGAATGCGC |  |  |  |
| *bla*_OXA_ | ACACAATACATATCAACTTCGC |  | 53 |  |
|  | AGTGTGTTTAGAATGGTGATC |  |  |
| *16S rRNA* | GGTAGTCYAYGCMSTAAACG | 263 | 62 |  |
|  | GACARCCATGCASCACCTG |  |  |
