# Supplementary material for: Development of aminoglycoside and β-lactamase resistance among intestinal microbiota of swine treated with lincomycin, chlortetracycline, and amoxicillin
Source: Front Microbiol. 2014 Nov 4;5:580. doi: 10.3389/fmicb.2014.00580 (PMC4219486; doi:10.3389/fmicb.2014.00580)
Supplement: Supplementary file 2 [file Table_2.DOC]

**Table S2.** Relative abundances of ARGs in fecal samples isolated before and after treatment, normalized to ambient 16S rRNA gene copies

| ARGs | Samples | | | |
| --- | --- | --- | --- | --- |
|  | A | B | C | D |
| *aac(3')-IIc* | 2.23E-01 | ***5.86E-01*** | 1.17E-01 | ***2.32E-02*** |
| *aadA1* | 1.19E-02 | ***1.02E-01*** | ***3.85E-02*** | 6.48E-03 |
| *aadB* | 2.20E+01 | 3.16E+01 | ***1.81E+02*** | ***9.37E+01*** |
| *aph(3')-II* | 1.07E-02 | ***3.67E-02*** | ***6.25E-02*** | ***3.06E-02*** |
| *aph(3')-IV* | 8.07E-01 | ***5.28E+00*** | ***5.03E+00*** | ***2.03E+00*** |
| *aph(4')-Ia* | 6.42E-01 | ***2.34E+01*** | ***1.01E+01*** | ***4.35E+00*** |
| *armA* | 9.31E+01 | ***1.28E+03*** | ***8.43E+02*** | ***4.25E+02*** |
| *aac(6')-Ib-cr* | 6.48E-01 | 6.60E-01 | ***2.93E+00*** | ***2.36E+00*** |
| *oqxA* | 1.62E+00 | 1.26E+00 | 2.64E+00 | ***5.47E-01*** |
| *bla*_CTX-M-9G_ | 3.17E-02 | ***9.21E-02*** | 6.94E-02 | ***1.95E-03*** |
| *bla*_TEM_ | 1.30E-02 | 2.29E-02 | 5.88E-03 | ***1.43E-04*** |
| *bla*_OXA_ | 1.19E-01 | ***1.73E+00*** | ***4.67E-01*** | ***1.32E-02*** |
| *ermA* | 4.63E+00 | ***2.65E+01*** | ***1.64E+02*** | ***4.05E+02*** |
| *ermB* | 1.92E+00 | ***1.35E+01*** | ***1.76E+01*** | ***2.08E+01*** |
| *mefA* | 2.62E-01 | ***1.79E+01*** | ***1.52E+01*** | ***8.75E+00*** |
| *lnuA* | 1.06E+01 | ***7.25E+01*** | ***1.64E+02*** | ***3.15E+02*** |
| *lnuF* | 3.53E+00 | ***8.98E+02*** | ***1.52E+03*** | ***1.13E+03*** |
| *ereA* | 1.31E+01 | ***3.10E+04*** | ***1.45E+04*** | ***2.84E+03*** |
| *tet*(L) | 1.58E-01 | ***2.36E+00*** | ***2.51E+00*** | ***7.07E-01*** |
| *tet*(Q) | 1.10E-01 | ***4.92E+00*** | ***1.47E+00*** | ***4.54E-01*** |
| *tet*(W) | 1.71E-01 | ***9.32E+00*** | ***2.91E+00*** | ***1.09E+00*** |
| gen^a^ | 1.17E+02 | ***1.34E+03*** | ***1.04E+03*** | ***5.25E+02*** |
| cip^b^ | 2.27E+00 | 1.92E+00 | 5.57E+00 | 2.91E+00 |
| amp^c^ | 1.64E-01 | ***1.84E+00*** | ***5.42E-01*** | ***1.53E-02*** |
| lin^d^ | 2.06E+01 | ***1.01E+03*** | ***1.87E+03*** | ***1.87E+03*** |
| ery^e^ | 1.99E+01 | ***3.11E+04*** | ***1.47E+04*** | ***3.27E+03*** |
| tet^f^ | 4.39E-01 | ***1.66E+01*** | ***6.90E+00*** | ***2.26E+00*** |

In all cases the standard deviation for triplicate samples was less than 15% of the mean.

The bold values in italics indicate statistical significance (P <0.05) between relative copies of ARGs isolated from fecal samples the day 0 before treatment (A) and the day 3, 6, 12 after treatment (B, C and D).

^a^ Sum of 7 aminoglycoside ARGs (*aac(3')-IIc*, *aadA1*, *aadB*, *aph(3')-II*, *aph(3')-IV*, *aph(4')-Ia* and *armA*)

^b^ Sum of 2 PMQR ARGs (*aac(6')-Ib-cr* and *oqxA*)

^c^ Sum of 3 β-lactam ARGs (*bla*_CTX-M-9G_, *bla*_TEM_ *and bla*_OXA_ )

^d^ Sum of 4 lincomycin ARGs (*ermA*, *ermB*, *lnuA* and *lnuF*)

^e^ Sum of 4 macrolide ARGs (*ermA*, *ermB*, *mefA* and *ereA*)

^f^ Sum of 3 tetracycline ARGs (*tet*(L), *tet*(Q) and *tet*(W))
