# Supplementary material for: Development of aminoglycoside and β-lactamase resistance among intestinal microbiota of swine treated with lincomycin, chlortetracycline, and amoxicillin
Source: Front Microbiol. 2014 Nov 4;5:580. doi: 10.3389/fmicb.2014.00580 (PMC4219486; doi:10.3389/fmicb.2014.00580)
Supplement: Supplementary file 3 [file Table_3.DOC]

**Table S3**. Correlations between relative abundances of different ARGs.

|  | ARGs | | | | | | | | | | | | |
| --- | --- | --- | --- | --- | --- | --- | --- | --- | --- | --- | --- | --- | --- |
| ARGs | *aac(3')-IIc* | *aadA1* | *aadB* | *aph(3')-II* | *aph(3')-IV* | *aph(4')-Ia* | *armA* | *aac(6')-Ib-cr* | *oqxA* | *bla*_CTX-M-9G_ | *bla*_TEM_ | *bla*_OXA_ | *ermA* |
| *aac(3')-IIc* | 1 | .912* | -.421 | -.027 | .524 | .817 | .673 | -.561 | -.174 | .682 | .952* | .933* | -.559 |
| *aadA1* | 0.912* | 1 | -.062 | .369 | .812 | .973** | .901* | -.214 | -.120 | .766 | .786 | .997** | -.334 |
| *aadB* | -.421 | -.062 | 1 | .865 | .532 | .100 | .333 | .959** | .131 | -.054 | -.567 | -.126 | .554 |
| *aph(3')-II* | -.027 | .369 | .865 | 1 | .818 | .487 | .653 | .746 | .261 | .410 | -.180 | .303 | .289 |
| *aph(3')-IV* | .524 | .812 | .532 | .818 | 1 | .888* | .963** | .385 | -.044 | .607 | .326 | .772 | .060 |
| *aph(4')-Ia* | .817 | .973** | .100 | .487 | .888* | 1 | .970** | -.018 | -.241 | .653 | .632 | .966** | -.112 |
| *armA* | .673 | .901* | .333 | .653 | .963** | .970** | 1 | .219 | -.240 | .572 | .455 | .881* | .051 |
| *aac(6')-Ib-cr* | -.561 | -.214 | .959** | .746 | .385 | -.018 | .219 | 1 | -.057 | -.297 | -.735 | -.267 | .762 |
| *oqxA* | -.174 | -.120 | .131 | .261 | -.044 | -.241 | -.240 | -.057 | 1 | .540 | .040 | -.166 | -.555 |
| *bla*_CTX-M-9G_ | .682 | .766 | -.054 | .410 | .607 | .653 | .572 | -.297 | .540 | 1 | .727 | .739 | -.679 |
| *bla*_TEM_ | .952* | .786 | -.567 | -.180 | .326 | .632 | .455 | -.735 | .040 | .727 | 1 | .809 | -.780 |
| *bla*_OXA_ | .933* | .997** | -.126 | .303 | .772 | .966** | .881* | -.267 | -.166 | .739 | .809 | 1 | -.340 |
| *ermA* | -.559 | -.334 | .554 | .289 | .060 | -.112 | .051 | .762 | -.555 | -.679 | -.780 | -.340 | 1 |
| *ermB* | -.095 | .236 | .717 | .677 | .635 | .452 | .618 | .795 | -.501 | -.183 | -.387 | .211 | .807 |
| *mefA* | .484 | .781 | .523 | .777 | .974** | .893* | .973** | .434 | -.238 | .458 | .240 | .750 | .239 |
| *lnuA* | -.442 | -.178 | .619 | .410 | .230 | .052 | .220 | .798 | -.568 | -.562 | -.694 | -.190 | .985** |
| *lnuF* | -.050 | .325 | .854 | .869 | .784 | .516 | .700 | .845 | -.238 | .048 | -.309 | .280 | .636 |
| *ereA* | .817 | .978** | .133 | .525 | .909* | .995** | .971** | -.009 | -.159 | .707 | .648 | .966** | -.168 |
| *tet*(L) | .308 | .460 | .101 | .209 | .471 | .613 | .629 | .233 | -.797 | -.130 | .025 | .481 | .598 |
| *tet*(Q) | .835 | .890* | -.204 | .158 | .647 | .913* | .825 | -.230 | -.499 | .446 | .646 | .913* | -.043 |
| *tet*(W) | .826 | .892* | -.178 | .183 | .663 | .920* | .837 | -.205 | -.496 | .447 | .632 | .913* | -.026 |
| gen^a^ | .591 | .852 | .437 | .727 | .982** | .937* | .994** | .324 | -.212 | .541 | .366 | .825 | .114 |
| cip^b^ | -.550 | -.248 | .824 | .751 | .264 | -.180 | .002 | .727 | .644 | .143 | -.535 | -.319 | .202 |
| amp^c^ | .934* | .998** | -.129 | .307 | .770 | .961** | .874 | -.277 | -.132 | .762 | .818 | .999** | -.366 |
| lin^d^ | -.204 | .156 | .829 | .761 | .629 | .372 | .564 | .886* | -.361 | -.169 | -.474 | .119 | .800 |
| ery^e^ | .812 | .976** | .140 | .530 | .912* | .996** | .974** | .001 | -.167 | .699 | .639 | .963** | -.155 |
| tet^f^ | .767 | .847 | -.143 | .186 | .647 | .896* | .825 | -.143 | -.565 | .362 | .552 | .869 | .076 |

|  | ARGs | | | | | | | | | | | | | |
| --- | --- | --- | --- | --- | --- | --- | --- | --- | --- | --- | --- | --- | --- | --- |
| ARGs | *ermB* | *mefA* | *lnuA* | *lnuF* | *ereA* | *tet*(L) | *tet*(Q) | *tet*(W) | gen | cip | amp | lin | ery | tet |
| *aac(3')-IIc* | -.095 | .484 | -.442 | -.050 | .817 | .308 | .835 | .826 | .591 | -.550 | .934* | -.204 | .812 | .767 |
| *aadA1* | .236 | .781 | -.178 | .325 | .978** | .460 | .890* | .892* | .852 | -.248 | .998** | .156 | .976** | .847 |
| *aadB* | .717 | .523 | .619 | .854 | .133 | .101 | -.204 | -.178 | .437 | .824 | -.129 | .829 | .140 | -.143 |
| *aph(3')-II* | .677 | .777 | .410 | .869 | .525 | .209 | .158 | .183 | .727 | .751 | .307 | .761 | .530 | .186 |
| *aph(3')-IV* | .635 | .974** | .230 | .784 | .909* | .471 | .647 | .663 | .982** | .264 | .770 | .629 | .912* | .647 |
| *aph(4')-Ia* | .452 | .893* | .052 | .516 | .995** | .613 | .913* | .920* | .937* | -.180 | .961** | .372 | .996** | .896* |
| *armA* | .618 | .973** | .220 | .700 | .971** | .629 | .825 | .837 | .994** | .002 | .874 | .564 | .974** | .825 |
| *aac(6')-Ib-cr* | .795 | .434 | .798 | .845 | -.009 | .233 | -.230 | -.205 | .324 | .727 | -.277 | .886* | .001 | -.143 |
| *oqxA* | -.501 | -.238 | -.568 | -.238 | -.159 | -.797 | -.499 | -.496 | -.212 | .644 | -.132 | -.361 | -.167 | -.565 |
| *bla*_CTX-M-9G_ | -.183 | .458 | -.562 | .048 | .707 | -.130 | .446 | .447 | .541 | .143 | .762 | -.169 | .699 | .362 |
| *bla*_TEM_ | -.387 | .240 | -.694 | -.309 | .648 | .025 | .646 | .632 | .366 | -.535 | .818 | -.474 | .639 | .552 |

| *bla*_OXA_ | .211 | .750 | -.190 | .280 | .966** | .481 | .913* | .913* | .825 | -.319 | .999** | .119 | .963** | .869 |
| --- | --- | --- | --- | --- | --- | --- | --- | --- | --- | --- | --- | --- | --- | --- |
| *ermA* | .807 | .239 | .985** | .636 | -.168 | .598 | -.043 | -.026 | .114 | .202 | -.366 | .800 | -.155 | .076 |
| *ermB* | 1 | .763 | .897* | .946* | .418 | .764 | .375 | .397 | .674 | .264 | .188 | .984** | .430 | .467 |
| *mefA* | .763 | 1 | .403 | .844 | .893* | .653 | .718 | .735 | .991** | .169 | .741 | .732 | .898* | .740 |
| *lnuA* | .897* | .403 | 1 | .751 | -.001 | .676 | .084 | .102 | .283 | .221 | -.216 | .885* | .012 | .200 |
| *lnuF* | .946* | .844 | .751 | 1 | .513 | .577 | .313 | .337 | .770 | .483 | .267 | .972** | .523 | .383 |
| *ereA* | .418 | .893* | -.001 | .513 | 1 | .537 | .873 | .880* | .942* | -.117 | .963** | .352 | 1.000** | .848 |
| *tet*(L) | .764 | .653 | .676 | .577 | .537 | 1 | .768 | .776 | .610 | -.370 | .453 | .638 | .547 | .838 |
| *tet*(Q) | .375 | .718 | .084 | .313 | .873 | .768 | 1 | 1.000** | .762 | -.520 | .900* | .238 | .874 | .993** |
| *tet*(W) | .397 | .735 | .102 | .337 | .880* | .776 | 1.000** | 1 | .776 | -.499 | .900* | .262 | .881* | .994** |
| gen | .674 | .991** | .283 | .770 | .942* | .610 | .762 | .776 | 1 | .102 | .818 | .637 | .945* | .769 |
| cip | .264 | .169 | .221 | .483 | -.117 | -.370 | -.520 | -.499 | .102 | 1 | -.304 | .430 | -.115 | -.499 |
| amp | .188 | .741 | -.216 | .267 | .963** | .453 | .900* | .900* | .818 | -.304 | 1 | .101 | .961** | .853 |
| lin | .984** | .732 | .885* | .972** | .352 | .638 | .238 | .262 | .637 | .430 | .101 | 1 | .363 | .328 |
| ery | .430 | .898* | .012 | .523 | 1.000** | .547 | .874 | .881* | .945* | -.115 | .961** | .363 | 1 | .851 |
| tet | .467 | .740 | .200 | .383 | .848 | .838 | .993** | .994** | .769 | -.499 | .853 | .328 | .851 | 1 |

Values indicate the Pearson correlation coefficient (r).

*p <0.05, **p <0.01.

^a^ Sum of 7 aminoglycoside ARGs (*aac(3')-IIc*, *aadA1*, *aadB*, *aph(3')-II*, *aph(3')-IV*, *aph(4')-Ia* and *armA*)

^b^ Sum of 2 PMQR ARGs (*aac(6')-Ib-cr* and *oqxA*)

^c^ Sum of 3 β-lactam ARGs (*bla*_CTX-M-9G_, *bla*_TEM_ *and bla*_OXA_ )

^d^ Sum of 4 lincomycin ARGs (*ermA*, *ermB*, *lnuA* and *lnuF*)

^e^ Sum of 4 macrolide ARGs (*ermA*, *ermB*, *mefA* and *ereA*)

^f^ Sum of 3 tetracycline ARGs (*tet*(L), *tet*(Q) and *tet*(W))

Aminov R.I., Garrigues-Jeanjean N. and Mackie R.I. 2001. Molecular ecology of tetracycline resistance: development and validation of primers for detection of tetracycline resistance genes encoding ribosomal protection proteins. Appl Environ Microbiol 67:22-32.

Bach H.J., Tomanova J., Schloter M. and Munch J.C. 2002. Enumeration of total bacteria and bacteria with genes for proteolytic activity in pure cultures and in environmental samples by quantitative PCR mediated amplification. J Microbiol Methods 49:235-45.

Bozdogan B., Berrezouga L., Kuo M.S. et al., . 1999. A new resistance gene, linB, conferring resistance to lincosamides by nucleotidylation in Enterococcus faecium HM1025. Antimicrob Agents Chemother 43:925-9.

Brinas L., Moreno M.A., Zarazaga M. et al., . 2003. Detection of CMY-2, CTX-M-14, and SHV-12 beta-lactamases in Escherichia coli fecal-sample isolates from healthy chickens. Antimicrob Agents Chemother 47:2056-8.

Cattoir V., Poirel L., Rotimi V., Soussy C.J. and Nordmann P. 2007a. Multiplex PCR for detection of plasmid-mediated quinolone resistance qnr genes in ESBL-producing enterobacterial isolates. J Antimicrob Chemother 60:394-7.

Cattoir V., Weill F.X., Poirel L., Fabre L., Soussy C.J. and Nordmann P. 2007b. Prevalence of qnr genes in Salmonella in France. J Antimicrob Chemother 59:751-4.

Cavaco L.M., Hasman H., Xia S. and Aarestrup F.M. 2009. qnrD, a novel gene conferring transferable quinolone resistance in Salmonella enterica serovar Kentucky and Bovismorbificans strains of human origin. Antimicrob Agents Chemother 53:603-8.

Costa D., Poeta P., Saenz Y. et al., . 2006. Detection of Escherichia coli harbouring extended-spectrum beta-lactamases of the CTX-M, TEM and SHV classes in faecal samples of wild animals in Portugal. J Antimicrob Chemother 58:1311-2.

De Graef E.M., Decostere A., De Leener E., Goossens H., Baele M. and Haesebrouck F. 2007. Prevalence and mechanism of resistance against macrolides, lincosamides, and streptogramins among Enterococcus faecium isolates from food-producing animals and hospital patients in Belgium. Microbial Drug Resistance 13:135-41.

Doi Y. and Arakawa Y. 2007. 16S ribosomal RNA methylation: emerging resistance mechanism against aminoglycosides. Clinical Infectious Diseases 45:88-94.

Haenni M., Saras E., Chaussiere S., Treilles M. and Madec J.Y. 2011. ermB-mediated erythromycin resistance in Streptococcus uberis from bovine mastitis. Vet J 189:356-8.

Kehrenberg C., Schwarz S., Jacobsen L., Hansen L.H. and Vester B. 2005. A new mechanism for chloramphenicol, florfenicol and clindamycin resistance: methylation of 23S ribosomal RNA at A2503. Mol Microbiol 57:1064-73.

Kim H.B., Park C.H., Kim C.J., Kim E.C., Jacoby G.A. and Hooper D.C. 2009a. Prevalence of plasmid-mediated quinolone resistance determinants over a 9-year period. Antimicrob Agents Chemother 53:639-45.

Kim H.B., Wang M., Park C.H., Kim E.C., Jacoby G.A. and Hooper D.C. 2009b. oqxAB encoding a multidrug efflux pump in human clinical isolates of Enterobacteriaceae. Antimicrob Agents Chemother 53:3582-4.

Lina G., Quaglia A., Reverdy M.E., Leclercq R., Vandenesch F. and Etienne J. 1999. Distribution of genes encoding resistance to macrolides, lincosamides, and streptogramins among staphylococci. Antimicrob Agents Chemother 43:1062-6.

Luthje P. and Schwarz S. 2007. Molecular basis of resistance to macrolides and lincosamides among staphylococci and streptococci from various animal sources collected in the resistance monitoring program BfT-GermVet. Int J Antimicrob Agents 29:528-35.

Malbruny B., Werno A.M., Murdoch D.R., Leclercq R. and Cattoir V. 2011. Cross-resistance to lincosamides, streptogramins A, and pleuromutilins due to the lsa(C) gene in Streptococcus agalactiae UCN70. Antimicrob Agents Chemother 55:1470-4.

Ng L.K., Martin I., Alfa M. and Mulvey M. 2001. Multiplex PCR for the detection of tetracycline resistant genes. Mol Cell Probes 15:209-15.

Park C.H., Robicsek A., Jacoby G.A., Sahm D. and Hooper D.C. 2006. Prevalence in the United States of aac(6')-Ib-cr encoding a ciprofloxacin-modifying enzyme. Antimicrob Agents Chemother 50:3953-5.

Singh K.V., Weinstock G.M. and Murray B.E. 2002. An Enterococcus faecalis ABC homologue (Lsa) is required for the resistance of this species to clindamycin and quinupristin-dalfopristin. Antimicrob Agents Chemother 46:1845-50.

Sutcliffe J., Grebe T., Tait-Kamradt A. and Wondrack L. 1996. Detection of erythromycin-resistant determinants by PCR. Antimicrob Agents Chemother 40:2562-6.

Tait-Kamradt A., Davies T., Cronan M., Jacobs M.R., Appelbaum P.C. and Sutcliffe J. 2000. Mutations in 23S rRNA and ribosomal protein L4 account for resistance in pneumococcal strains selected in vitro by macrolide passage. Antimicrob Agents Chemother 44:2118-25.

Xia L.N., Li L., Wu C.M. et al., . 2010. A survey of plasmid-mediated fluoroquinolone resistance genes from Escherichia coli isolates and their dissemination in Shandong, China. Foodborne Pathog Dis 7:207-15.

Yan J.J., Hong C.Y., Ko W.C. et al., . 2004. Dissemination of blaCMY-2 among Escherichia coli isolates from food animals, retail ground meats, and humans in southern Taiwan. Antimicrob Agents Chemother 48:1353-6.
